# Development and validation of the participation in treatment decision-making scale for adults with malocclusion (PTDMS-AM)

**DOI:** 10.1186/s12903-025-06825-2

**Published:** 2025-10-11

**Authors:** Xiangying Hu, Bixia Wang, Ting Pan, Weijun Yuan, Lili Hou

**Affiliations:** 1 Department of Nursing, Shanghai Ninth People’s Hospital, School of Medicine, Shanghai Jiao Tong University, Shanghai, China; 2 Department of Oral & Cranioï¿¿maxillofacial Surgery, Shanghai Ninth People’s Hospital, College of Stomatology, National Center for Stomatology, Shanghai Jiao Tong University School of Medicine, Shanghai Jiao Tong University, National Clinical Research Center for Oral Diseases, Shanghai, China

**Keywords:** Malocclusion, Development, Scale, Patient participation, Decision-making

## Abstract

**Background:**

Participation in decision-making is crucial for patients with malocclusion. It is important to assess the extent to which patients are actively involved in such decision-making. Therefore, this study developed an instrument that evaluates patient participation in treatment decision-making among adults with malocclusion and tested its reliability and validity.

**Methods:**

A cross-sectional instrument-development methodological approach was adopted. Guided by the concept of participation and shared decision-making theory, an initial scale was developed through literature analysis, qualitative interviews, expert evaluation, and a pre-survey. From September to December 2023, 257 patients from three tertiary general hospitals in Shanghai were selected for a questionnaire survey, item analysis, and exploratory factor analysis. From January to May 2024, 269 patients from these hospitals were selected to conduct a questionnaire survey for confirmatory factor analysis and criterion-related validity.

**Results:**

The final scale included 21 items across three dimensions. The Cronbach’s α coefficient was 0.953; split-half reliability, test-retest reliability, and the scale level content validity index were 0.957, 0.885, and 0.926 respectively. The correlation coefficients between each dimension and the total score of the scale and SDM-9 scores were 0.590–0.650 (*P* < 0.05). Exploratory factor analysis extracted three common factors; the Kaiser-Meyer-Olkin value was 0.974 and the Bartlett’s sphericity test χ^2^ value was 5652.33 (*P* < 0.001); the cumulative variance explained was 60.920%. The fit indices of the scale model tested in the confirmatory factor analysis were as follows: χ^2^/df = 1.059, RMR = 0.033, GFI = 0.936, CFI = 0.968, TLI = 0.964, NFI = 0.641, RMSEA = 0.015. Confirmatory factor analysis revealed that the scale’s factor structure was stable.

**Conclusions:**

This instrument is a reliable and valid measurement tool for assessing adult patients’ level of participation in decision-making regarding treatment for malocclusion.

**Supplementary Information:**

The online version contains supplementary material available at 10.1186/s12903-025-06825-2.

##  Background

Malocclusion is a major oral disease affecting facial aesthetics and function. Data from the 2017 National Oral Health Survey revealed that the prevalence in China is 74%, which means that 1,037 million people experience some degree of facial deformities. In China, the proportion of people receiving malocclusion treatment is only one-sixth that in the United States. Further, malocclusion in adults is more serious than in minors. Current treatments for adults primarily include orthodontic treatment and orthognathic surgery combined with pre- and post-surgical orthodontic treatment to correct jaw positional disorders and malocclusion, restore function, and improve aesthetics [1]. However, the cost of orthognathic surgical treatment, lengthy orthodontic treatment course, and variety of combined orthognathic and orthodontic treatment options pose a challenge to adult patients’ treatment decisions.

Patients are gradually becoming involved in the decision-making process regarding malocclusion treatment options [2] following the advancement of technological methods used in orthognathic surgery and orthodontic treatment modalities, the development of digitalization [3], and the visualization of treatment options [4, 5]. Malocclusion treatment is a low-certainty medical decision, and adult patients should be encouraged to optimally fulfill their complex roles and responsibilities in the decision-making process. Simultaneously, clinical decisions made individually by a dental practitioner may lead to suboptimal treatment and poor patient outcomes [6]. Therefore, patient participation is important for successful treatment.Participation is a proactive behavior. Patient participation is a multidimensional concept that encompasses cognitive, emotional, and behavioral dimensions that represent the degree of behavioral, emotional, and intellectual efforts made in treatment [7–10]. In treatment decision-making, patient participation revolves around patients’ rights and opportunities to influence and engage in decision-making regarding their care through dialogue attuned to their preferences, potential, and a combination of their experiential and professional expert knowledge [11, 12]. This is consistent with the shared decision-making (SDM) theory proposed by Charles [13]. Shared decision-making requires the activation of both healthcare providers and patients. It is a collaborative approach in which patients are encouraged to share their preferences, values, and goals and actively engage in discussions and decisions about their treatment options [14]. All of these features are manifestations of patient participation. Thus, the patients’ cognitive, emotional, and behavioral activities should be a sub-element in evaluating patient participation.

Patient participation assessment tools play an important role in related research. Based on different understandings of the connotations of decision-making participation and its structure, diverse decision-making participation assessment tools exist. Of these tools, the nine-item Shared Decision-Making Questionnaire (SDM-Q-9) [15], perceived involvement in care scale (PICS) [16], and CollaboRATE can evaluate the participation in treatment decision-making from the perspective of the patient. The SDM-Q-9 focuses on assessing the level of patient participation in decision-making, providing a comprehensive evaluation of shared decision-making processes. The PICS is a brief self-administered measure of patients’ perceptions of doctor patient communication during medical encounters. It consists of a three-part assessment of patient perceived physician behaviors that facilitate participation in decision-making, information exchange, and patient decision-making. In contrast, CollaboRATE is a rapid assessment tool composed of only three items, developed in collaboration with patients [17]. The SDM-Q-9 and CollaboRATE were both compiled based on SDM-related theories; however, CollaboRATE exhibits a stronger ceiling effect [18]. Patient participation evaluation tools have been developed internationally, but related research in China remains in its infancy.

Decision-making participation is very important for patients with malocclusion, being related to patient satisfaction with treatment programs and cooperation, which in turn facilitates patient empowerment. If patients do not actively participate in treatment decision-making, they may not be fully aware of the long-term benefits and potential risks of the different treatment options. This lack of awareness can lead to suboptimal treatment. For instance, some patients may choose a less invasive treatment option without fully understanding that it may not be the most effective treatment for complex malocclusions. If patients do not understand the reasons underlying the treatment plan due to a lack of participation, they may not follow post-treatment instructions properly, which could cause a relapse of malocclusion. Therefore, improving patient participation in decision-making should be prioritized. Although there are existing tools can measure patient participation levels, considering the methodological quality of existing scales and the cultural differences between China and other countries, it is necessary to develop localized assessment tools. Therefore, this study developed an instrument to measure the degree of patient participation in treatment decision-making among adults with malocclusion.

## Methods

### Design

This methodological study was conducted according to the scale-development process of Costa Palacio and Danielle [19].

### Phase I: scale development

####  Initial item generation

To identify the initial items of the instrument, we consulted literature published between 2000 and 2022 to analyze the characteristics of SDM-related theories and participation concepts.

We also selected for interviews adult patients with malocclusion who participated in treatment decisions. The interviews were conducted face-to-face from January 26 to March 4, 2023, and each interview lasted 30–45 min. Using purposive sampling, we reached data saturation after 13 interviews as no additional themes emerged. The main questions for the patient interviews were, “What was your main experience in decision-making participation?” “How did you participate in treatment decision-making?” and “Why did you actively participate in your decision-making?” Traditional content analysis was used to analyze the interview data [20]. The attributes of patient participation identified through the literature review and interviews were cognitive participation, specific decision-making participation behavior, and the emotional perceptions of patients during the decision-making process.

The initial scale was generated based on the shared decision-making (SDM) theory proposed by Charles [13]. Guidance was provided through a conceptual framework of patient participation. The item pool was constructed by referring to existing scales [9, 15–17, 21–23], reviewing interview data, and brainstorming; a total item pool of 35 entries was formed.

Expert discussions and revisions were conducted of the 35 initial items. In the first evaluation, seven experts were invited to screen and supplement the items of the scale, including nurses and doctors, who modified the expression of two items, merged three items, deleted one item with a general meaning, added one item, and retained 32 items. In the second round of expert evaluation, eight experts were invited to evaluate the relevance and clarity of the expression of the items: one item was revised and 32 items were retained (8 for cognitive participation, 16 for behavioral participation, and 8 for emotional participation sub-dimensions).

#### Content validity

An expert panel evaluated the content validity of the draft instrument. The panel consisted of three clinical nurses, two orthodontic experts, three orthognathic experts, and one psychological expert, each of whom had more than 10 years of experience. The experts scored the relevance of each item based on their own experience, using a four-point scale (1 = irrelevant, 2 = somewhat relevant, 3 = relevant, 4 = very relevant). The item-level content validity index (I-CVI) was obtained by dividing the number of experts who assigned scores of 3 and 4 for the scale items by the total number of experts. The scale-level content validity index (S-CVI) is equal to the average I-CVI for each item. I-CVI > 0.780 [24] and S-CVI ≥ 0.800 [25] were used as the criteria for the scale to be considered valid.

#### Preliminary survey

The preliminary instrument was assessed for appropriateness of grammar, vocabulary, and similar matters. Thirty adult patients with malocclusion from a tertiary general hospital in Shanghai who had participated in decision-making programs within one month were selected using convenience sampling. The respondents reported that the content of the items was reasonable and easy to understand and that the time to complete the instrument was less than 6 min, without modification or supplementation.

### Phase 2. Validity and reliability

####  Participants

This study was conducted at three hospitals in Shanghai, China. The participant selection criteria were as follows: (1) adults (age ≥ 18 years), (2) diagnosed with malocclusion, (3) had made treatment decisions within the previous month. The exclusion criteria included an inability to communicate or difficulties filling out the questionnaire. Questionnaires with more than 5% missing values were excluded from the analysis.

According to the principle of scale design, the sample size should be 5–10 times the number of scale items [26], the predefined required sample size for this study was determined to be 160–320. Considering a predicted 10% dropout rate, a minimum of 176 participants was required for exploratory factor analysis.

#### Survey instruments

The instruments used consisted of the following: (1) A general information questionnaire that consisted of 12 items that collected information such as age, sex, and marital status. (2) The first draft of the Patient Participation in Treatment Decision-Making Scale. (3) The Chinese version of the SDM-Q-9. The SDM-Q-9 [15] was revised from the Shared Decision-Making Questionnaire (SDM-Q) [27] with nine items and Sinicized by Luo [28]. The Cronbach’s α coefficient of the Chinese version was 0.946, and the structure of the modified scale was consistent with that of the original scale. The Cronbach’s α coefficient of the scale as used in this study was 0.930.

#### Data collection

Anonymous surveys of individuals were conducted from September to December 2023, using convenience sampling to recruit clinical patients from three tertiary general hospitals in Shanghai. The inclusion and exclusion criteria were the same as those used in the pre-test survey. Questionnaires were distributed to participants who provided informed consent by members of the unified training group. The data were collected using mobile phones. The participants completed the questionnaire by themselves. Questionnaires were immediately recovered and examined by the researchers. A total of 265 questionnaires were distributed in the reliability and validity test survey, of which 257 valid questionnaires were recovered, for an effective recovery rate of 96.98%.

#### Item analysis

The critical ratio method, Cronbach’s α coefficient, and correlation coefficients were used to screen items. The criteria were as follows: (1) Questionnaires were arranged in ascending order of total score, with the top and bottom 27% set as the high and low score groups, respectively. An independent samples t-test was used to compare the two groups. Items whose scores did not differ significantly between groups were deleted. (2) Items with a correlation coefficient < 0.400 with the total score of the scale were excluded. (3) If the total Cronbach’s α coefficient of the scale increased significantly after an item was removed, then the item was deleted.

#### Validity test

Exploratory factor analysis (EFA) was used to evaluate construct validity. Prior to conducting EFA, Kaiser–Meyer–Olkin (KMO) and Bartlett’s sphericity tests were used to determine the suitability of the items for the analysis. EFA was performed using principal components analysis with orthogonal rotation. The evaluation criteria were as follows: cumulative variance explained > 50%, eigenvalue > 1, and factor loading ≥ 0.400 [29, 30]. Double-loading items, that is, if the loading value of the same item on two or more common factors was > 0.400 and the difference between the loadings was < 0.200, were deleted. Criterion-related validity was tested by analyzing the correlation between the newly developed instrument and the SDM-Q-9. *P* < 0.05 was considered statistically significant.

####  Reliability

The internal consistency of the scale was measured using the Cronbach’s α coefficient and split-half reliability. Cronbach’s α coefficient > 0.700 was used as the criterion for evaluating the overall reliability of the scale [31]. Split-half reliability was greater than 0.7, indicating that the scale items had good reliability. To assess test-retest reliability, 14 participants who volunteered for retesting completed the questionnaire again, 2 weeks after their initial responses.

#### Confirmatory factor analysis

The sample size for confirmatory factor analysis (CFA) should be larger than that for EFA, and at least 200 samples are needed [26]. From May to August 2024, a convenience sampling method was adopted to conduct a questionnaire survey of clinical patients in three tertiary general hospitals in Shanghai using the general information questionnaire, the newly developed instrument, and the SDM-Q-9. The inclusion and exclusion criteria were the same as those used in the pre-test survey. A total of 300 questionnaires were distributed, of which 269 valid questionnaires were returned for an effective recovery rate of89.6%. The evaluation criteria for the CFA were as follows [32]: chi-square/degrees of freedom (χ^2^/df) < 5; goodness-of-fit index (GFI), comparative fit index (CFI), normed fit index (NFI), Tucker-Lewis index (TLI), and incremental fit index (IFI) all > 0.900; root mean-square error of approximation (RMSEA) < 0.080; and root mean square residual (RMR) < 0.05.

####  Data analysis

The data were analyzed using SPSS 25.0 and AMOS 25.0. Measurement data were described by mean ± standard deviation, and count data were described by frequency and percentages. Item analysis, reliability testing, and structural validity analysis were conducted on the scale data from the first round of the survey. Validation factor analysis and correlation validity analysis were conducted using the data from the second round of the survey. Statistical significance was considered to be *P*<0.05.

####  Ethical considerations

This study was approved by the Ethics Committee of Shanghai Ninth People’s Hospital (approval no. SH9H-2023-T411-1). Written and mobile consent was obtained from all participants who understood the study’s purpose and voluntarily agreed to participate. Data were collected through interviews and surveys.

## Results

### Characteristics of the participants

The reliability and validity test investigated the responses of 257 patients (76 males, 29.57%; 181 females, 70.42%). The average age was 25.19 ± 4.45 years (range: 18–39 years). Of the participants, 214 (83.26%) were unmarried, 39 (15.17%) were married, and 4 (1.55%) were divorced; 185 participants (71.9%) had a bachelor’s degree or higher level of education.

CFA was performed on the data of 269 patients (92 males, 34.2%; 177 females, 65.79%). The average age was 26.13 ± 4.96 years (range: 18–43 years). Of the participants, 219 (81.41%) were unmarried and 50 (18.58%) were married. There were 216 participants (80.2%) who had a bachelor’s degree or higher.

### Item analysis

The correlation coefficients between all items and the total score of the scale were > 0.400. Therefore, no items were deleted.The deletion of any given item did not lead to a significant increase in Cronbach’s α coefficient. There were statistically significant differences between items in the high and low subgroups (*P* < 0.05). Therefore, all 32 items were retained.

### Construct validity

According to the results of the EFA, the KMO value was 0.974, and the Bartlett’s sphericity test χ^2^ value was 5652.33 (*P* < 0.001). This suggested that the data were suitable for factor analysis. A total of three factors were extracted; the loading of each item on the corresponding factor was ≥ 0.400, with a cumulative variance explained of 60.920%.

The theoretical model and the loading values of the items were considered. Nine items that exhibited double-loading characteristics were deleted, leaving 21 items across the three dimensions: cognitive participation (5 items), behavioral participation (10 items), and emotional participation (6 items; Table 1).

**Table 1:** Exploratory factor analysis results of the participation in treatment Decision-Making scale for adults with malocclusion

Item number	Dimension/Item	% of Variance (Cumulative)	Eigenvalues	Com^*^	Factor		
					F1	F2	F3
	Cognitive participation	50.888 (50.888)	16.028				
1	I understand what the doctor is saying			0.575	0.652	0.324	0.212
4	I relate the information provided by the doctor to information I previously gathered			0.651	0.739	0.190	0.263
5	I remember the details of treatment options			0.643	0.736	0.214	0.235
6	I can recognize the advantages and disadvantages of different treatment options			0.664	0.720	0.338	0.179
7	I ask questions when I have them			0.631	0.725	0.298	0.131
	Behavioral participation	6.854 (57.742)	2.193				
8	I actively search for information about treatments (online or from patients) to better understand treatment options			0.655	0.401	0.693	0.120
10	I know about the doctor (doctor’s surgical style, aesthetic habits)			0.594	0.328	0.662	0.222
11	I prepare questions to ask			0.523	0.065	0.664	0.278
13	I listen calmly and attentively			0.600	0.276	0.648	0.322
14	I repeat key information in my own words to confirm understanding			0.559	0.168	0.598	0.416
15	I respond to physician-initiated questions about my thoughts on treatment options			0.581	0.119	0.648	0.384
19	I clearly express desired results (e.g., ideal facial shape)			0.698	0.293	0.754	0.244
20	I consult the doctor about their recommendations (e.g., which treatment plan they prefer)			0.704	0.297	0.732	0.282
21	I discuss the treatment plan with the doctor			0.722	0.290	0.752	0.269
23	I reach consensus on the treatment plan with the doctor			0.532	0.205	0.613	0.349
	Emotional participation	3.178 (60.920)	1.017				
24	I am interested in treatment options and like to participate actively in the discussion and selection of treatment options			0.706	0.288	0.257	0.746
25	I feel excited when I learn something about dentistry while participating in the discussion			0.526	0.338	0.423	0.483
26	I feel a sense of accomplishment when I establish a cooperative relationship with the doctor			0.589	0.276	0.404	0.591
29	I feel involved when the doctor recognizes my suggestions			0.626	0.225	0.283	0.704
30	I feel fulfilled when questions that are of great concern to me are answered by the doctor			0.560	0.248	0.462	0.534
31	I don’t think it’s necessary to participate in the discussion (decision-making process), just to know the doctor’s decision			0.536	0.390	0.369	0.497

* Communalities

### Content validity and criterion-related validity

The S-CVI value was 0.926 and the I-CVI ranged from 0. 813 to 1.000, which were greater than the standard values. The correlation coefficients between the scores of each dimension and the total score of the scale and the total score of the SDM-Q-9 ranged from 0.590 to 0.650 (*P* < 0.05).

### Reliability analysis

The Cronbach’s α coefficient of the total scale was 0.953, those of the three dimensions were 0.878, 0.909, 0.860, respectively. The overall split-half reliability was 0.957, and the split-half reliabilities for each dimension were 0.856, 0.866, and 0.856, respectively. The test-retest reliability value was 0.885.

### Confirmatory factor analysis

**Fig. 1:**
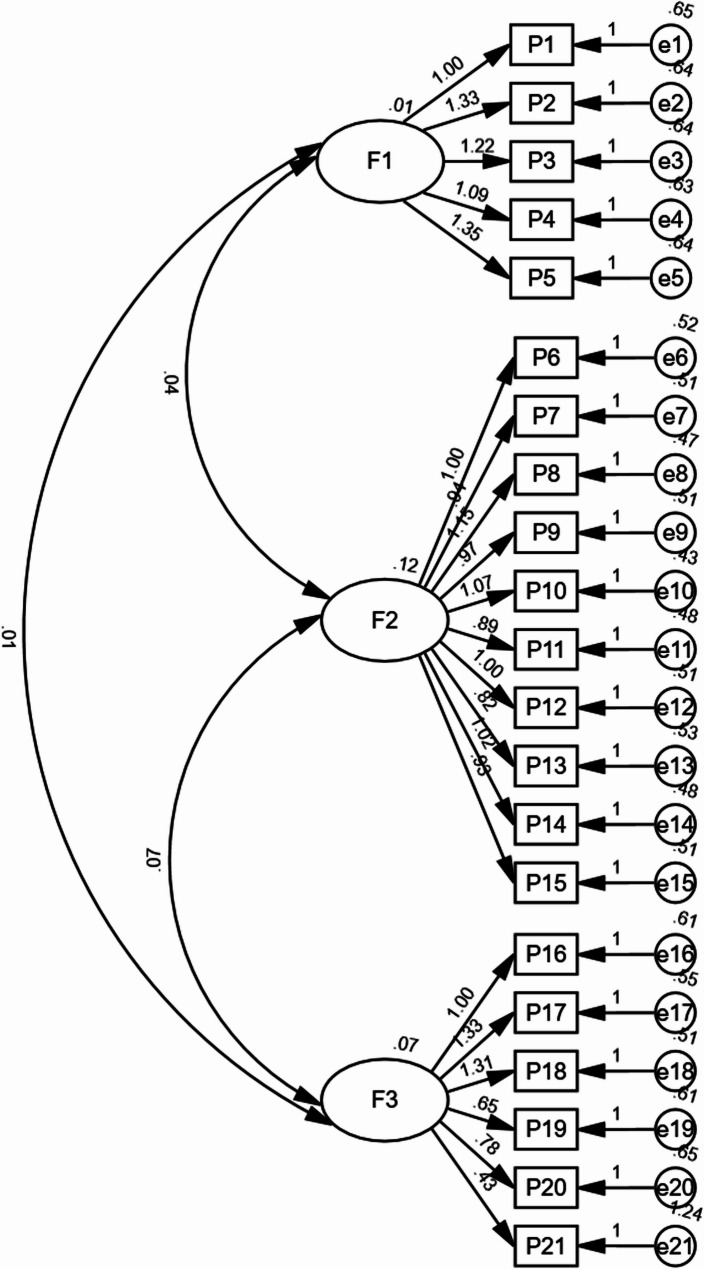
Path diagram of the final model of the Participation in Treatment Decision-Making Scale for Adults with Malocclusion

The results of CFA revealed the following fit statistics: χ^2^/df = 1.059, GFI = 0.936, CFI = 0.968, TLI = 0.964, NFI = 0.641, RMSEA = 0.015, and RMR = 0.033. Except for NFI, which was close to the fit standard, the remaining indices reached the criterion values, indicating that the model fit the data well. The structure of the draft scale was confirmed using 3 factors and 21 items (Fig. 1).

### Comparison of EFA and CFA factor loadings

In the EFA, the factor loadings ranged from 0.483 to 0.754. In the CFA, the factor loadings ranged from 0.146 to 0.506. Table 2 presents a comparison of the factor loadings obtained in the EFA and CFA.

**Table 2:** Comparison of EFA and CFA factor loadings

Item	EFA Factor Loading	CFA Standardized Loading	Difference
1	0.652	0.146	0.506
4	0.739	0.194	0.545
5	0.736	0.179	0.557
6	0.720	0.160	0.560
7	0.725	0.197	0.528
8	0.693	0.434	0.259
10	0.662	0.419	0.243
11	0.664	0.506	0.158
13	0.648	0.427	0.221
14	0.598	0.497	0.101
15	0.648	0.408	0.240
19	0.754	0.439	0.315
20	0.732	0.362	0.370
21	0.752	0.454	0.298
23	0.613	0.414	0.199
24	0.746	0.312	0.434
25	0.483	0.419	0.064
26	0.591	0.425	0.166
29	0.704	0.210	0.494
30	0.534	0.242	0.292

### Scoring method of the participation in treatment Decision-Making scale for adults with malocclusion

The newly developed scale items are evaluated using a five-point Likert scale: 1 point (Strongly Disagree), 2 points (Disagree), 3 points (Undecided), 4 points (Agree), and 5 points (Strongly Agree); the final item is reverse-scored. The total score range is 21–105 points. Higher scores indicate higher levels of patient participation.

## Discussion

The Participation in Treatment Decision-Making Scale for Adults with Malocclusion(PTDMS-AM) was developed using a rigorous scientific approach. Through a literature review and based on the concept of participation and the SDM theory proposed by Charles [13], this study focused on adult patients with malocclusion in China, explored their decision-making participation ability, and compiled a tool to assess the decision-making participation ability of such patients. This study provided the initial draft scale through expert verification and a preliminary survey.

The development process was meticulous. The initial scale draft was formed through expert verification and a preliminary survey. In the two expert verification rounds, the experts provided constructive suggestions to ensure scale reliability. This study then conducted two rounds of questionnaire surveys to test the scale’s reliability and validity, and CFA, ultimately forming a formal scale of Participation in Treatment Decision-Making for Adults with Malocclusion. The development process of the scale was rigorous and standardized, ensuring scientific validity.

Notably, the factor loadings in the PTDMS-AM differed significantly between EFA and CFA, particularly for the cognitive participation dimension, for which the CFA loadings were generally lower than those in the EFA. This discrepancy aligns with the methodological differences between EFA and CFA highlighted by Hair et al. [33]. EFA permits cross-loadings, whereas CFA requires items to load exclusively on their designated factors, which may result in lower factor loadings in CFA. These differences highlight the need for further validation of the scale in diverse populations to ensure its robustness and applicability. While the sample sizes for EFA (*n* = 257) and CFA (*n* = 269) were adequate for preliminary validation, larger and more diverse samples would strengthen the generalizability of the results.

Additionally, the NFI value of 0.641 of the PTDMS-AM approached, but did not fully meet, the fit standard (> 0.900). This could be attributed to sample characteristics or model specifications. Despite these issues, the PTDMS-AM exhibited good reliability and validity, and the overall factor structure remained consistent between the EFA and CFA, supporting the robustness of the three-factor model. Three factors extracted from 21 items validated in EFA explained 60.9% of the total variance, indicating acceptable construct validity. In the CFA, the fit indices of the model were all within the acceptable range, and the fit to the data was good.

The Chinese version of the SDM-9 was used as this study’s criterion correlation index; the large correlation coefficients between the PTDMS-AM and SDM-9 indicated that the former scale is relatively ideal. The reliability of the PTDMS-AM reliability was high, with a Cronbach’s α value of 0.953.The Cronbach’s α coefficient of each dimension was between 0.860 and 0.909, the split-half reliability value was 0.957, and the test-retest reliability value was 0.885; thus, the scale had good internal consistency.

The PTDMS-AM has good clinical practicability. Currently, the SDM-9 scale modified by Kriston et al. [15] is widely used [34–37], but lacks specificity for adults with malocclusion. The content of existing assessment tools only involves the objective participation behavior of patients, and does not involve the subjective perceptions of patients during the participation process.

Participation in treatment decision-making requires patients to have strong abilities in information acquisition, communication, information reception and balancing, as well as emotional perception. Accordingly, this scale evaluates three dimensions—cognitive participation, action participation, and emotional participation—which reflect the specific elements of patient participation in a more targeted way. This multi-dimensional approach is in full alignment with the conceptual framework of patient participation proposed by Castro [11].

By offering deeper insights into patients’ cognitive, behavioral, and emotional engagement, the PTDMS-AM can assist clinicians in better understanding patients’ perspectives, enabling the development of more personalized treatment plans. Ultimately, this approach has the potential to enhance treatment outcomes and improve patient satisfaction.

In addition, the time to complete the scale was less than 6 min, indicating that the items of the scale were easy to understand, the number of items was appropriate, items were accepted by the respondents, and the scale had good operability.

This study was conducted only in tertiary hospitals in Shanghai, which may not strictly represent the entire adult population with malocclusion in China. Factors such as patient selection, hospital characteristics, and socioeconomic differences could further influence the outcomes and their applicability to broader contexts. Therefore, the scale needs to be tested and validated on a larger scale, including with more diverse populations and settings to enhance the generalizability of the findings.

The behavior of patients participating in treatment decision-making ceases after determining the treatment plan. When respondents use this scale for self-assessment, they generally need to complete it through recall, which inevitably lead to some degree of recall bias. Participants may overestimate or underestimate their behaviors or outcomes, leading to inaccuracies in the reported results. When conditions permit, the recall period should be shortened to the extent possible, by applying the scale shortly after the patient has made the treatment decision.

The PTDMS-AM was designed to capture patients’ self-reported perceptions of their involvement. The absence of an external assessment by treating dentists or orthognathic surgeons limits our ability to evaluate the correlation between patients’ self-reported participation and their actual participation as observed by clinicians. This could affect the clinical validity of the questionnaire, as self-reported measures may be influenced by patients’ subjective interpretations or social desirability bias. Future studies should consider incorporating external validation measures, such as clinician assessments of patient participation; this could provide additional insights into the scale’s clinical applicability and robustness.

## Conclusion

The scale developed in this study includes 3 dimensions and 21 items; all indicators met the requirements for a valid and reliable scale. The PTDMS-AM is a scientific evaluation tool for measuring the level of decision-making participation in adult patients with malocclusion. It is easily used to investigate the level of decision-making participation in different regions and in different hospitals, thereby providing a reference for promoting shared decision-making interventions.

Although there were differences in the factor loadings and fit indices compared to existing literature, its multidimensional design and strong psychometric properties make the PTDMS-AM an effective tool for evaluating patient participation. Future research should further validate the PTDMS-AM across diverse cultural and clinical contexts and incorporate external assessments by clinicians to enhance its clinical validity.

## Supplementary Information

Supplementary Material 1.

## Data Availability

The datasets used during the current study are available from the corresponding author on reasonable request.

## Abbreviations

SDM: Shared Decision Making, a collaborative process where patients and healthcare providers work together to make informed healthcare decisions based on clinical evidence and patient preferences
