# Supplementary material for: Development and validation of the participation in treatment decision-making scale for adults with malocclusion (PTDMS-AM)
Source: BMC Oral Health. 2025 Oct 11;25:1602. doi: 10.1186/s12903-025-06825-2 (PMC12515386; doi:10.1186/s12903-025-06825-2)
Supplement: Supplementary file 1 — Supplementary Material 1. [file 12903_2025_6825_MOESM1_ESM.pdf]

Appendix：

Participation in Treatment Decision-Making Scale for Adults with Malocclusion (PTDMS-AM)

The purpose of this questionnaire is to allow you to describe your level of involvement in the decision-making process for malocclusion treatment. When answering the questions, please reflect on your cognitive, behavioral, and emotional participation in the treatment decision-making process. "Strongly agree" means that the description generally applies to you and you consider it to be correct or appropriate, while "strongly disagree" indicates that you believe it to be incorrect or inappropriate. The questionnaire uses a Likert 5-point scale, ranging from "strongly disagree," "disagree," "neutral (undecided)," "agree," to "strongly agree," with corresponding scores of 1 to 5. Please mark "√" on the number that best describes your feeling.

Item number	Item	options				
		strongly agree	agree	neutral (undecided)	disagree	strongly disagree
1	I understand what the doctor is saying	5	4	3	2	1
2	I relate the information provided by the doctor to information I previously gathered	5	4	3	2	1
3	I remember the details of treatment options	5	4	3	2	1
4	I can recognize the	5	4	3	2	1

	advantages and disadvantages of different treatment options					
5	I ask questions when I have them	5	4	3	2	1
6	I actively search for information about treatments (online or from patients) to better understand treatment options	5	4	3	2	1
7	I know about the doctor (doctor's surgical style, aesthetic habits)	5	4	3	2	1
8	I prepare questions to ask	5	4	3	2	1
9	I listen calmly and attentively	5	4	3	2	1
10	I repeat key information in my own words to confirm understanding	5	4	3	2	1
11	I respond to physician-initiated questions	5	4	3	2	1

	about my thoughts on treatment options					
12	I clearly express desired results (e.g., ideal facial shape)	5	4	3	2	1
13	I consult the doctor about their recommendations (e.g., which treatment plan they prefer)	5	4	3	2	1
14	I discuss the treatment plan with the doctor	5	4	3	2	1
15	I reach consensus on the treatment plan with the doctor	5	4	3	2	1
16	I am interested in treatment options and like to participate actively in the discussion and selection of treatment options	5	4	3	2	1
17	I feel excited when I learn something about dentistry while participating in the	5	4	3	2	1

	discussion					
18	I feel a sense of accomplishment when I establish a cooperative relationship with the doctor	5	4	3	2	1
19	I feel involved when the doctor recognizes my suggestions	5	4	3	2	1
20	I feel fulfilled when questions that are of great concern to me are answered by the doctor	5	4	3	2	1
21	I don't think it's necessary to participate in the discussion (decision-making process), just to know the doctor's decision	5	4	3	2	1
